# Supplementary material for: Impact of Human Papillomavirus-Negative Dominance in Oropharyngeal Cancer on Overall Survival: A Population-Based Analysis in Germany from 2018 to 2020
Source: Cancers (Basel). 2023 Nov 2;15(21):5259. doi: 10.3390/cancers15215259 (PMC10650408; doi:10.3390/cancers15215259)
Supplement: Supplementary file 1 [file cancers-15-05259-s001.zip › cancers-2646752-supplementary.pdf]

Supplementary Table S1

Supplementary Table S1. Distribution of head and neck cancer patients according to clinical and demographic parameters

**Parameter**	**Frequency (N)**	**%**
All	498	100
Year of diagnosis		
2018	162	32.5
2019	176	35.3
2020	160	32.1
Tumor registry region		
Jena	95	19.1
Erfurt	174	34.9
Gera	69	13.9
Nordhausen	60	12.0
Suhl	100	20.1
	**Mean±SD**	**Median, Range**
Age on diagnose, years	62.45 ± 9.5	61, 33-95
Follow-up (months)	20.49 ± 14.85	20, 0-52
Follow-up of patients alive (months)	23.56 ± 14.75	24, 0-52

Supplementary Table S2

Supplementary Table S2. Univariable analyses on predictors for overall survival

**Parameter**		**Mean ± SD**	**2-Year OS**	**95%-CI**	**p**
OS		40.87 ± 0.94	79.72%	39.02-42.71	
Gender	Male	39.77 ± 1.11	77.81%	37.60-41.95	**0.040**
	Female	44.42 ± 1.68	86.09%	41.13-47.72	
Age	≤ 61 years	41.46 ± 1.26	79.77%	38.99-43.92	0.527
	> 61 years	40.28 ± 1.41	79.67%	37.52-43.05	
Cigarette smoking	Yes	37.71 ± 1.37	75.39%	35.02-40.41	**0.004**
	No	44.03 ± 1.24	85.12%	41.60-46.46	
Alcohol drinking	Yes	37.44 ± 1.71	75.29%	34.10-40.79	**0.017**
	No	43.02 ± 1.12	83.45%	40.83-45.21	
HPV-Status	HPV +	47.18 ± 1.08	90.86%	45.07-49.29	**<0.001**
	HPV -	36.29 ± 1.35	73.61%	33.65-38.94	
T classification	T1	45.47 ± 1.68	88.17%	42.18-48.76	**<0.001**
	T2	45.28 ± 1.47	86.72%	42.42-48.14	
	T3	39.16 ± 1.84	78.51%	35.56-42.76	
	T4	31.93 ± 2.01	70.34%	28.00-35.86	
N classification	N0	45.34 ± 1.45	87.79%	42.51-48.17	**<0.001**
	N1	45.61 ± 1.71	86.81%	42.25-48.96	
	N2	37.27 ± 1.58	75.61%	34.18-40.36	
	N3	30.32 ± 2.90	67.74%	24.64-36.00	
M classification	M0	41.94 ± 0.94	81.54%	40.09-43.78	**<0.001**
	M1	26.61 ± 3.73	62.50%	19.29-33.92	
Cancer staging	I	49.32 ± 1.16	96.34%	47.05-51.60	**<0.001**
	II	46.89 ± 1.39	90.91%	44.17-49.60	
	III	41.36 ± 1.94	83.87%	37.57-45.16	
	IV	31.55 ± 1.73	67.50%	28.16-34.94	
Grading	G1	34.35 ± 4.39	70.59%	25.76-42.95	0.955
	G2	40.30 ± 1.35	79.13%	37.65-42.95	
	G3	40.08 ± 1.81	78.32%	36.52-43.63	
	undifferentiated	39.94 ± 3.38	80.56%	33.32-46.56	
Primary surgery	Yes	45.56 ± 0.97	86.59%	43.67-47.45	**<0.001**
	No	31.85 ± 1.58	70.97%	28.76-34.94	
Radiation	Yes	43.00 ± 0.97	83.12%	41.11-44.89	**<0.001**
	No	33.28 ± 2.52	67.59%	28.34-38.22	
Chemotherapy	Yes	42.73 ± 1.13	82.35%	40.52-44.94	**0.015**
	No	38.57 ± 1.60	75.98%	35.42-41.71	
Immunotherapy	Yes	34.40 ± 2.12	71.91%	30.25-38.56	0.064
	No	41.88 ± 1.02	81.48%	39.88-43.87	
Recurrence	Yes	39.98 ± 2.40	75.36%	35.29-44.68	0.771
	No	41.05 ± 1.02	80.42%	39.05-43.06	

OS – overall survival; CI – confidence interval
